# Does the Norwegian Police Force Need a Well-Functioning Combat Mindset?

**DOI:** 10.3389/fpsyg.2020.01075

**Published:** 2020-06-03

**Authors:** Ole Boe, Glenn-Egil Torgersen, Tom Hilding Skoglund

**Affiliations:** ^1^USN School of Business, Department of Business, Strategy, and Political Sciences, University of South-Eastern Norway, Drammen, Norway; ^2^Psychology Department, Bjørknes University College, Oslo, Norway; ^3^USN School of Business, Department of Business, History and Social Sciences, Center for Security, Crisis Management and Emergency Preparedness, University of South-Eastern Norway, Horten, Norway

**Keywords:** combat mindset, unforeseen situations, stress, coping with stress, police: military, combat mindset training

## Abstract

This article discusses the apparent lack of a well-functioning combat mindset evident in the Norwegian Police Force during the terrorist attacks in Norway on July 22, 2011. We describe what a well-functioning combat mindset is and then continue to discuss challenges linked to the current mindset in the Norwegian Police Force. We then elaborate upon how the experience of an acute stress reaction may affect one’s ability to solve a mission. Then we describe how to cope with stress and the importance of a well-functioning combat mindset, and we then discuss techniques in order to build a well-functioning combat mindset. Furthermore, we describe how coping with an extreme cognitive load and a well-functioning combat mindset are related. Finally, we suggest a method for practicing combat mindset (CM-training). The combat mindset training needs to include both realistic training and systematic reflection in order to a better ability to deal with sudden and unforeseen events.

## Introduction

*The Spartans do not ask how many are the enemy but where are they* (Plutark cited in Pressfield, 2011).

July 22—for Norwegians, mentioning this date leaves a certain bitter aftertaste in the mouth, much like mentioning 9/11 for an American. On Friday, July 22, 2011, Norway was hit by two terrorist attacks (SNL, 2017). The first one was the bomb attack on the government quarter, and the second one was the mass murder at Utøya. Both terrorist attacks were conducted by the Norwegian citizen Anders Behring Breivik. The two incidents were the worst terrorist attacks in Norway after World War II. Seventy-seven people were killed in the attacks. Many were severely injured, and it also resulted in significant material damage. In terms of mental strain and cognitive load, serious repercussions for those affected still remain.

In the aftermath of the attacks, a lot of focus naturally turned to the police work executed. Police work is composed of several elements, including leadership, teamwork, interaction and communication with the regular population, and handling terror incidents. Each aspect contributes to the successful execution of operative tasks (Meld St. 21,2012; Johannessen, 2013). The importance of these different elements increases with time pressure.

In many police operations, as in military operations, it is important that operators are able to swiftly form an understanding of the situation and still maintain a dynamic situational awareness, thus enabling them to make decisions rapidly. Situational awareness and decision making are the key processes dictating what kind of effects the methods the police use will have on targets and on the resolution of the situation at hand. It is therefore of utmost importance to understand the factors that may weaken situational awareness, how to regain awareness, and even how to better utilize situational awareness (Gustavsberg, 2018).

The traditional method of analyzing police operations does not, in retrospect, usually focus on mental and cognitive functions. Police operations analyses have basically been concentrated on law, tactics, technology, and management but not on mental and cognitive matters (Hess et al., 2014). It becomes, therefore, important to shed more light on all relevant information, including mental and cognitive factors, to avoid the risk of a continued lack of knowledge-based management in the police force.

Based on our former studies, which focused on learning and interaction under risky and unforeseen incidents, our main conclusion was that a combat mindset is of great import when it comes to coping with complex and unpredictable situations (Torgersen, 2015, 2018; Boe and Ingdahl, 2017; Boe and Torgersen, 2018; Yanilov and Boe, 2020). The present article therefore takes these findings a step further, leading to the following research question: Does the Police need a well-functioning combat mindset?

The authors are aware of the risk of the bias of hindsight when judging previous events. It is easy to say after a crisis what preparations, priorities, and actions that should or should not have been done. Our intention is not to criticize the job performance of the individuals participating in the July 22, 2011 police operation, as we do not have detailed information on those matters. The purpose of this paper is rather to argue for the relevance of what we call a combat mindset in such extreme events. We will point out that a combat mindset increases the individual’s operational functioning when encountering unpredictable and threatening circumstances, making adequate aggression control and the neutralization of dangerous enemies more likely.

As a starting point for discussing this question, we will use the experiences from the incident at Utøya on July 22, 2011, and discuss how the Norwegian Police Force handled this incident. These are difficult questions to answer. There are no indisputable solutions. Central here is the role of the police and the formal political directives for handling these types of incidents. However, this is more related to a strategic and political level, and our focus in this article is restricted to discussing the level of skills and abilities of the individual police officer. When the focus is on individual skills and abilities in operative police work, it is vital to understand the issued standard operational procedures (SOP) that are guided by law, policy, and directives. Also, although we focus on *the concept of individual resilience*, this is a distinct but related concept from *organizational resilience*. Organizational resilience is founded on the resilience of the individuals comprising the organization, and their work performance contributes to the organization’s *effectiveness and productivity* (Gustavsberg, 2018).

Gustavsberg (2018) also discusses that law enforcement officers in their right minds wish to resolve these difficult situations successfully, according to laws, rules, and regulations in a humane way. However, in these situations it may not be the willpower of an officer that dictates the outcome of the situation. In this article we solely deal with individual resilience, which may be seen as an important precursor to a later well-functioning combat mindset (Yanilov and Boe, 2020). Achieving better situational awareness and improved performance especially when required to make on-the-spot decisions on use of force is an important topic in order to be able to handle these types of situation.

Some factors stand out, and the most important is the one that concerns having a well-functioning “combat mindset.” Much of what happened before, during and after the event at Utøya on July 22 deals with the different involved actors and their combat mindset, or lack thereof. In Norwegian, one often uses the term “*kampvilje*” (fighting will), where willpower is an important part of one’s mental attitude. For reasons of simplicity, the concept of combat mindset will be used in this article. One definition of the term combat mindset is the following: “Willingness and ability to continue the fight despite the high levels of mental and physical pain” (Boe, 2006, 2007, author’s translation).

In the July 22 Commission’s report, a conclusion was made that the Norwegian Police operation at Utøya was too slow and that leadership was lacking (NOU, 2012, p. 14). Furthermore, it was estimated in the same report that the police may have lost as much as 25 min because of the confusion that followed (NOU, 2012, p. 14). In the book Police Culture by Johannessen (2013), it is argued that there is no doubt that the police could have acted more quickly and that the events were characterized by weak decision-making based on group-thinking, subsequent tunnel vision, as well as lack of creativity.

This article is based on the authors’ understanding of the events of July 22, without the authors themselves having been involved in the operation. The authors also do not have access to the full tactical reasoning that underlies the decisions made by different elements of the Norwegian Police during the incident at Utøya. The first author has worked more than 20 years in the Norwegian Armed Forces (NAF), including several years of service in one of Norway’s special military units as an officer and 15 years as an instructor at the Norwegian Military Academy and the Norwegian Defence Command and Staff College. The second author has worked as a lecturer at the Norwegian Police University College for 4 years and as an organizational psychologist in the NAF for 7 years focusing on the selection of high-risk operational personnel. The last author has worked for more than 25 years in the NAF, mainly within educational facilities in the NAF, dealing with military pedagogics and doing research on unforeseen incidents and how to handle them better. The suggestions made in this article are thus based on known theories and research on the topics of stress, stress management, and how to develop a well-functioning combat mindset. Many people have been concerned about the response time of the Norwegian Police Force in connection with the action conducted at Utøya (NOU, 2012, p. 14). One central question in this article is therefore to discuss how to train a well-functioning combat mindset and how this mindset should work so as to shorten the response time when facing this type of events.

In this article, we first describe what a well-functioning combat mindset is. Our starting point is a narrative from a Norwegian military officer serving on an international operation in Afghanistan during the same time period as the incidents took place at Utøya in 2011. This narrative is at the extreme end of what a well-functioning combat mindset can be for a military officer. We then continue discussing the major challenges linked to current mindset of the Norwegian Police Force on July 22, 2011. Following this line of thought, we then elaborate upon how experiencing a stress reaction may affect a person’s ability to solve a mission. Thereafter, we deal with how to cope with stress and the importance of a well-functioning combat mindset. We then describe the techniques that work best in order to build a well-functioning combat mindset. Furthermore, we describe the relationship between coping with an extreme cognitive load and a well-functioning combat mindset. Finally, we suggest a method for practicing a combat mindset, referred to as CM-training. Finally, we draw some conclusions as to how a well-functioning combat mindset can be implemented for the Norwegian Police Force.

## Some of the Norwegian Police Force Challenges Linked to the Current Mindset on July 22

There is no doubt that many things went wrong for the Norwegian Police on July 22. A quick review of the Gjørv Commission’s report from July 22 clearly shows this (NOU, 2012, p. 14). In the Northern Buskerud Police District (NBPD), the Operations Officer was alone on duty. Eventually, the Operations Officer tried to call the operations center in the capital of Norway, Oslo, but they received no answer. A good mobilization of a number of police officers was conducted, but this was because these police officers happened to be available by phone. The rubber boat in the basement of the police station in the city of Hønefoss lacked both air and gasoline. This may be a sign in advance of a poor combat mindset and insufficient visualization of different possible emerging scenarios. There was a failure to update the daily logs and thus insufficient information to be handed over to the new Operations Officer. The result was that the new Operations Officer did not know the status of the situation on July 22. The Operations Officer in NBPD was not aware that the Norwegian Special Police were on their way in cars, which led to incorrect preparations. The Norwegian Special Police left a team to be picked up by helicopter at a place called Sollihøgda, which testifies to their having a good situation awareness and, thus, a well-functioning combat mindset. In this phase, the Norwegian Special Police were already doing a better job, probably because they had a much better combat mindset. This is also described well in the book by Stensønes (2017), who has followed the Norwegian Special Police over the course of 2 years.

Some of the challenges for the Norwegian Police Forces on July 22 may well stem from the NBPD’s new emergency network not yet having been developed, but the events may also have been a result of mental factors or a poor combat mindset. On the other hand, the same report (NOU, 2012, p. 14) shows that the Operations Officer in Southern Buskerud Police District (SBPD) decided without any request being made to send aid up to NBPD, which testifies to a better understanding of the situation. Patrols from SBPD were standing idle on arrival at the city of Hønefoss, but they quickly decided to go ahead on their own initiative to Storøya, an island situated 3.6 km (approximately 2.2 miles) from Utøya. When the various police officers later approached Utøya, it is likely that they found themselves in a situation that they had not foreseen earlier. This may have caused a great deal of stress for each individual police officer.

Previous research has shown that the ability to judge risk will change under stress. You will become more cautious and want to believe that things are more dangerous than they really are (Matthews et al., 2007, 2011). One will also in retrospect see a tendency to overestimate what one has done in relation to the mission (Matthews et al., 2011). This can be seen when one reads the Norwegian Police Force’s own report discussing the events that took place during the incident at Utøya on July 22, 2011, the not at all critical Sønderland report (Politidirektoratet, 2012). It may in itself be true that it was not possible to do anything else in the actual situation at Utøya on July 22, 2011, and we emphasize that our thinking here is based on our own experiences of educating and training police and military officers and not in relation to the incident at Utøya.

## What Is Meant by a Well-Functioning Combat Mindset?

Leaders and operators in law enforcement and the military know that a well-functioning combat mindset is important, but not many know how it can best be developed. Even in an elite military unit like the United States Navy Seals, it is described that no one really teaches you for instance how to focus, other than to say “focus!” (Machowicz, 2002). Being able to maintain focus on solving a task or assignment under stress is vital for many professions and not just for the military and police. The lack of focus is often caused by the stress reactions that can occur in such a situation as the one at Utøya. One challenge is to train to maintain correct focus in a difficult situation so that one develops a well-functioning combat mindset.

*Greater than any other calling, the life of the warrior requires mental skills in combination with physical or mechanical skills. Yet, mental training is an area which has been long neglected in the fields of conflict management and force application* (Doss, 2007, p. 65).

The problem is rather that things were not executed well enough in advance of what happened at Utøya on July 22, 2011. Or in other words, the Norwegian Police Force could certainly have spent more time and resources on developing a better combat mindset. In situations characterized by chaos and friction, where people involved may to varying degrees be paralyzed by fear, one still has to solve the mission. This will, among other things, require the ability to quickly adapt by having good situational awareness and significant real-life training prior to an incident taking place. In addition, the individual’s ability to visualize, i.e., to envision various possible situations and solutions, is important (Yanilov and Boe, 2020). These are factors that help build up an efficient combat mindset (Boe, 2006, 2011).

So, what is a well-functioning combat mindset? We will use a practical example from the military environment, but be aware that the described mindset may not be appropriate for most of the regular situations that the Norwegian Police Force will find themselves in. Take look at an interview we conducted with a Commander of the Norwegian Coastal Ranger Commandoes after having served on a military operation in Afghanistan in 2011. Below, the Commander describes what it means to have a well-functioning combat mindset in relation to the situation he found himself in:

We wanted to be seen as good representatives of the Provincial Reconstruction Team (PRT), the North Atlantic Treaty Organization (NATO) and our country through what we did. Our role model was the strategic corporal, and this had implications for how you appear and how you carry your guns. If you feel confident, it means that you carry your gun in a different way. Our choice of a softer profile made sure we were perceived as safe, with control. We did not focus on posture. The motto was “calm, relaxed and ready.” This resulted in a consciousness in the way of conducting yourself, while you at the same time kept thinking that you were a guest in Afghanistan. The consequence of this again leads to showing consideration. In addition, it was important to get out on the ground and start working. We worked particularly with the Afghan National Security Forces and police forces, and they were very thankful that we worked on establishing ourselves as a security actor to get the PRT in place. We showed ourselves as a functioning security actor and we supported the mission that was to establish a safe and secure Afghanistan. This meant that we were perceived as reliable and we easily became friends with people. Simultaneously our mindset made it easy to switch between being a friend and being completely “ruthless” when it was necessary. It is important to note that we did our very best to avoid conflicts. Meanwhile, there is no point being naive. We knew that there were many people out there who had an intent to kill us. Accepting it in every part of your body that there are people who will use any means to kill you or take you as prisoner is a special experience, I think. For us this meant to focus on what our job was and to be tactically wise. This meant for instance not to put ourselves in situations where we lost all control, when someone had placed improvised explosive devices, lying ready in ambush or tried to kill us with missiles while we slept. They can just try because that’s all they get the chance to do. They chose the wrong team to attack (authors’ translation).

This is how a Commander of the Norwegian Coastal Ranger Commandoes describes the way of thinking that he and his team had in Afghanistan in 2011. It is worth noting what he says about alternating between being a friend and being completely “ruthless” when it was necessary. This is the same mindset that we find in the writings of United States General Krulak (1999) describing the strategic corporal. Krulak points out that the complexity we are now experiencing in various operations means that you must be prepared to handle a wide range of tasks. This is where the concept of the strategic corporal comes in, i.e., practicing on the ability to quickly adapt to different situations. This is what is reflected in the mentality of the Commander and his team. The big question is whether this thinking has taken hold within the Norwegian Police Force. If not, what then? On the other hand, is this type of mindset necessary or even desirable for the Norwegian Police Force?

It is claimed that up to 90% of one’s performance can be traced to mental skills. It is rare that this is less than 40% (Asken et al., 2010). A major challenge that perhaps the Norwegian Police Force (and for that matter the NAF) suffer from, is that they are rarely instructed and trained in how to develop a well-functioning combat mindset. Doss (2007) describes that one of the most overlooked areas of training, which probably comes with the greatest number of limitations, is that of the development of a winning mindset or, using our terminology, a well-functioning combat mindset. A winning or combat mindset is simply a state of mind that ensures one’s survival during a critical incident. A winning or combat mindset is composed of several components. Among these components are situational awareness, concentration, anticipation, level headedness, and the maintenance of dexterity. Above all, the essence of a winning or combat mindset is self-control of your emotions and of your body.

A combat mindset is also described as a “survival mindset” (Siddle, 1995), “fighting spirit” (Boe, 2006), “mental toughness,” “grace under fire,” “nerves of steel” (Asken et al., 2010), or a “winning mindset” (Doss, 2007). Siddle(1995, p. 134) defines a well-functioning combat mindset as follows:

A survival mindset denotes a presence of mind allowing the warrior to focus completely on the task of the moment. It is a mindset void of emotion, where perception, analysis, and response merge into one process. The warrior enters a state where perceptions are more acute, actions becomes reflexive, and concentration is not hampered by the potential of death. Most importantly, the warrior attains the ability to concentrate in presence of death and act reflexively without hesitation.

Siddle (1995) uses the term survival mindset, but the authors’ understanding is that Siddle means the same as combat mindset. A discussion point might be if it is possible to be void of emotion, as indicated by Siddle. A well-functioning combat mindset consist of three parts: the mindset one has before, during, and after an event. Before an event occurs, you should be awake and relaxed. If there is time to be afraid, you should make a conscious effort to change this into controlled aggression. The reason for this is that the brain cannot easily tell the difference between fear and aggression, but it is easier to channel aggression than fear into something useful to solve a mission. This aggression must be controlled and directed toward resolving the ongoing mission. Aggression must be turned on and off as required (Boe and Ingdahl, 2017; Yanilov and Boe, 2020). Murray (2004) argues that to condition and develop aggression you need skills, stress control and the willingness to take lifes. A previous study of how to train aggression and aggression control in professional soldiers has shown this type of training to be very effective (Boe and Ingdahl, 2017). The willingness to kill can also be increased, and the role of the group and the aggressive predisposition of the individual soldier (or for that matter the individual police officer) are important factors here (Boe and Johannessen, 2015). The mindset during an event should be one of complete focus upon the techniques and tactics that you should use in order to solve the situation (Boe, 2006). The mindset after an event should be one where you feel relieved that is it over, grateful that it went well, and proud because you performed well (Boe, 2006).

A well-functioning combat mindset will also be a result of good tactical, technical, and mental skills (Yanilov and Boe, 2020). Key questions might then be: How can we develop an efficient combat mindset, and why would this have been relevant in the incident taking place at Utøya? This raises many questions, and one unfortunately finds no easy answers.

A more encompassing definition of combat mindset is that a well-functioning combat mindset consists of three elements. First, it includes attributes such as courage, determination, mental toughness, and aggression. Second, it includes relaxation and defusing of emotions such as fear, anger, antagonism, and frustration. Finally, a well-functioning combat mindset also includes focus, concentration, and self-control (Yanilov and Boe, 2020). These three areas are integrated, and there are also overlapping sections between them. Table 1 gives an overview of the elements of a well-functioning mindset.

**TABLE 1:** Elements included in a well-functioning combat mindset.

A state of mind that ensures one’s survival during a critical incident
It consists of several elements.
In general:
Situational awareness, concentration, anticipation, level headedness, and maintenance of dexterity
Self-control of emotions and body
Good tactical, technical, and mental skills
Courage, determination, mental toughness, and aggression
Relaxation and defusion of emotions such as fear, anger, antagonism, and frustration
Focus, concentration, and self-control
Before an incident:
Feeling awake and relaxed, turning an eventual fear into controlled aggression
During an incident:
Complete focus upon the techniques and tactics that you should use
in order to solve the situation
After an incident:
Feeling relief, gratitude, and pride

## The Major Challenge for the Norwegian Police Force

The challenges for United States law enforcement officials have been described well by the American police instructor Murray (2004). Murray claims that lack of a realistic training level is a critical challenge for the police in the United States. It is reasonable to believe that this also applies to the police in Norway. According to senior safety adviser Bjørn Egeli in the Norwegian Police Force, the police need education and training that makes them feel safe in such a dramatic situation as the one at Utøya (Dagbladet, 2011). The weapons training the police receive is restricted to 11 h of introduction to the new tactics “shooting in progress” and then a 40-h annual certification program (Dagbladet, 2011). Murray (2004) also claims that the regular police officer does not spend much time practicing the physical skills necessary to function in difficult situations. He believes that police officers do not put in enough time practicing emotional and psychological exercises to build a functioning combat mindset. Perhaps part of the explanation why the police did not have a sufficiently developed and well-functioning combat mindset at Utøya can be found in Murray’s thoughts on the lack of training of both physical and psychological skills. The Norwegian Police Force is supposed to engage in confidence-building tasks, aid-related tasks, civil justice tasks, management tasks, prisoner transport, and border control (Meld St. 42, 2004). This leaves little time to develop a well-functioning combat mindset that can be used in extreme situations.

This all calls into question whether Norway’s basic police educational period includes teaching and training relevant for the initial development of a combat mindset. Starting with a selection phase, upcoming police officers complete a 3-year bachelor’s degree in policing. While the Norwegian selection of police students is based on competencies like “power to act” and “maturity,” the selection methods used for mapping these competencies are suboptimal, as demonstrated in a predictive validity study (Skoglund,2018a, b). The police student suitability selection is based entirely on psychometric testing, an interview, and a group communication exercise; realistic job simulations with elements of stress activation are lacking. It is therefore the authors’ view that the initial selection process for police students is not particularly focused on personnel requirements related to high-stress environments. The educational period is relevant for developing a broad array of operational skills, as students attend several practical police exercises and a whole year working as a junior officer under the guidance of an experienced police officer. The educational period consists also of several academic subjects, such as psychology, sociology, criminology, and police history. Police union representatives have often problematized if the surpluses of academics have lessened the focus on practical and operational matters in law enforcement work (e.g., Bjerke Lilleåsen, 2017), which can indicate experiences of inadequate operational skills among newly educated operators. Events such as the terrorist attacks July 22 certainly actualizes such a question.

## How May Experiencing a Stress Reaction Affect One’s Ability to Solve a Mission?

It is often said that one is under stress or under pressure in different situations. This article does not distinguish between the concepts of stress and pressure. Stress is defined as the activation of the portion of the autonomic nervous system that is called the sympathetic nervous system. The body turns on and is ready for fight or flight (Cannon, 1932; Boe, 2006). Also occurring is tachypsychia, which is a neurological condition that alters the perception of time (Boe, 2006).

Stress can be considered an overload that weakens our ability to process information and solve problems, and it thus can have an effect on our performance when carrying out a mission. Simply put, one can say that the stress is the result of an imbalance between perceived competence and what the situation demands (Harris and Berger, 1983: Lazarus and Folkman, 1984). There is little doubt that the situation the Norwegian Police found themselves in regarding the terrorist attack carried out by Anders Behring Breivik at Utøya on July 22, 2011 was difficult. The situation probably demanded such a high level of competence that this must have been experienced as very uncomfortable. Much of this can perhaps be explained because the police simply had not anticipated that such a situation would arise. The result is that such a situation, once it does occur, becomes much more difficult to handle than if one had imagined similar possible situations before. The stress one may experience when one suddenly finds oneself in a situation like that at Utøya is the result of perception, and one’s perception can be changed through exercise; that is, both mental and physical exercise. An important point is that this training should be as realistic as possible and related to the context in which one operates (Murray, 2004). This was arguable the point of the much quoted sentence in the *On Combat* book by Grossman and Christensen (2008 p. 75), “…in combat you do not rise to the occasion, you sink to the level of your training.” With a well-functioning combat mindset, it is possible to reduce much of the perceived stress associated with solving a very unforeseen mission, such as the incident at Utøya. Reactions would probably also have been quicker if the police had trained on this type of scenario before the incident took place.

## Coping With Stress and the Importance of a Well-Functioning Combat Mindset

The issue of stress as an important focus for military research was identified as early as in 1917 (Yerkes, 1918). Even with all technological development we have had since then, the fact remains that maintaining an effective ability to solve missions in stressful situations will continue to be a challenge, and this is true also for the Norwegian Police, especially when technology fails in the way it did on July 22, 2011. The scope and magnitude of this problem has long been known, especially when it comes to performance in a combat situation (Marshall, 1947; Schwartz and Winograd, 1954). A summary made by the United States Army School of Advanced Military Studies concluded that combat stress will be one of the most significant factors behind the loss of human resources (Coomler, 1985).

Others have argued that in critical moments of a mission, one’s susceptibility to psychological threats could be a decisive factor in whether one succeeds or fails on a mission (Wherry and Curran, 1966). Previous research has shown that there are many different stressors that can affect one’s ability to solve a mission. These factors include sound (Broadbent, 1978), performance pressure (Baumeister, 1984), workload (Wickens, 1979), expected threat of shock (Wachtel, 1968), dangerous situations (Hammerton and Tickner, 1969; Burke, 1980), clearing bombs (Rachman, 1982), and combat stress (Williams, 1984).

It is likely that the police officers that participated in the operation at Utøya experienced several of the above stressors and were affected by this. These stressors have also been shown to increase physiological activation, thus leading to an increased heart rate, labored breathing, and tremors (Rachman, 1982). It has been shown that one’s ability to solve problems is lessened (Yamamoto, 1984) and that one is more rigid in the execution of a task under stress (Staw et al., 1981). Further research has shown that performance stress alone can increase the incidence of errors in operational procedures up to three times the normal number of errors (Villoldo and Tarno, 1984). An example of the rigidity or performance stress could be the Norwegian Special Police’s decision to put all their 11 men with full equipment in an inflatable boat registered for 10 people. The inflatable boat experienced an engine failure and took in water, and a civilian boat had to come to the rescue. It has also been shown that the time one uses to perform manual tasks doubles under stressful conditions (Idzikowski and Baddeley, 1983). Several of these stressors may have affected the police officers when trying to solve missions at Utøya.

## Which Techniques Work to Build a Well-Functioning Combat Mindset?

The learning mechanisms that have proven to work best to get soldiers to operate even under extreme stress in combat are known as classical and operant conditioning (Grossman, 1995). There is reason to assume that this will also apply to police officers. Classical and operant conditioning are psychological techniques used when one wants to train people to react correctly in emergency or combat situations.

Classical conditioning is a form of learning in which a person learns to connect a stimulus with response. Operant conditioning means, for example, to give a person a reward to reinforce an action (Weiten, 2001). In situations involving great stress, one’s past experiences, stress tolerance, and the degree of realistic training one had before ending up in a fight similar to the situation will affect how effectively one will be able to respond and function. These are important factors in the development of a functioning combat mindset. If you have practiced something enough times, then it is easier to do the same thing under stress. This is why you have different drills for different things. Part of the purpose of the drill is to counteract a possible paralysis that can occur due to stress (Boe et al., 2011, 2012). Through the drill, one practices the procedural memory, i.e., the memory for procedures or actions (Moldjord and Holen, 2005). On the other hand, the situation at Utøya was probably so extreme that it was probably too big a challenge for most people who were present to identify the actual extent of the situation. Precisely the ability to identify and recognize what the emerging situation means is an important part of a combat mindset (Sde-Or Lichtenfeld and Yanilov, 2001). The reason for this is that a correct perception of a threat situation might serve as a trigger. The result is that you will be able to activate an offensive combat mindset much faster than if you do not recognize the situation. In other words, in order to recognize a “worst-case scenario” and be able to do something about it, you must previously have thought about the “worst-case scenario.”

If one does not have the resources or time to train enough physically to solve a mission, an alternative solution is to practice more mentally. When you transit to a mission site, you should visualize the operation you are about to conduct (Doss, 2007). Mental training can never, however, replace realistic and physical training on how to solve a mission. An important method of developing an efficient combat mindset includes the ability to stop negative thoughts. By being aware of your own negative thoughts you can change them to become positive. At the same time, working on reducing anxiety, worries, and doubts in relation to one’s skills is also important. The statement “I will fix this” is an example of such a positive affirmation, and this practive may counteract the negative thoughts one has about oneself and one’s own performance in relation to solving a given mission. An example of the use of positive coping strategies is highlighted in a study that showed that a majority of the British military personnel being deployed in conjunction with the Falklands War in the 1980s used positive thinking as their primary coping strategy (Limbert, 2004). Whether the police officers on their way to Utøya made use of positive affirmations to stop negative thoughts is not known by the authors.

Another important method is to practice the so-called “combat” or “tactical breathing” (Asken et al., 2010). This could help lower the heart rate to a level where a person will function optimally. Research has shown that one could have a powerful increase in heart rate caused by hormone-induced activation of the nervous system. This heart rate increase is caused by perceived stress, uncertainty, fear, or being scared. At over 145 beats per minute, one’s ability to think clearly and to process information deteriorates. By taking control of one’s breathing, it can be possible to stay between 115 and 145 beats per minute (Siddle, 1995). In this zone, the brain and body function optimally together. This zone is known as the optimal survival and combat performance zone (Grossman and Christensen, 2008). An important part of one’s combat mindset is precisely the ability to control yourself so that you remain well within this zone. In other words, this involves taking control of your breathing when you begin to feel stress. The extent to which the police officers made use of various breathing techniques during the incident at Utøya is not known by the authors. Practicing tactical breathing can be combined with training with a lot of adrenaline and other stress hormones in the body, a training method referred to as Adrenaline Stress Conditioning (Boe, 2006). The purpose of Adrenaline Stress Conditioning is to move the threshold for where one fails to perform under hormone-induced stress, i.e., to work well even with heart rates higher than 145 beats per minute. The first objective is thus to practice so that one in a difficult situation remains between 115 and 145 heartbeats per minute. If the situation escalates and the heart rate increases to the point that you can no longer carry out a task, the goal then becomes to function effectively even with large amounts of adrenaline and hormones.

## Extreme Cognitive Load and a Well-Functioning Combat Mindset

Under extreme cognitive load one will lose the ability to think rationally (Siddle, 1995), and one’s ability to remember the sequence and duration of things will be gone. This is a phenomenon known as critical incident amnesia (Grossman and Siddle, 2001; Grossman and Christensen, 2008). When the heart rate rises above 175 beats per minute, this may occur. It is possible that some of the police officers at Utøya experienced this. Again, an important factor is to mentally have gone through possible scenarios in advance and to take advantage of various breathing techniques to take control of yourself in the situation.

One of the most difficult aspects of stressful and uncertain situations is the decision-making process (Von Schell, 1933). This is because we receive information at different times. As humans, we have an inherent tendency to want to wait a little longer. Maybe we can get some more information before we make a decision? A common strategy is to reduce the uncertainty one experiences by attempting to collect more information, which leads to a delayed action. This may therefore often involve a disadvantage in operational situations (Lipshitz, 1997).

Von Schell (1933) makes the point that it is usually more difficult to determine the actual time of making a decision than to formulate the decision. Practicing making quick decisions under great degree of uncertainty helps develop a well-functioning combat mindset. This is a mental skill. Anyone can learn any skill. But if the learned skill just becomes technical or physical, it is of little help or value. Working with the ability you (and possibly others) have to cope with stress requires a lot from you mentally. Developing a willingness to take lives requires even more mentally. To develop these three parts, that is the technical, the physical, and the mental part, is crucial in order to develop an aggressive mindset as part of a well-functioning combat mindset. Equally important will be to develop a culture of controlled aggression by channeling fear, anger, and anxiety over many hours of relevant real-world physical and mental training that is based on values (Howe, 2005). By values, we mean the will to do the right thing based on correct values in order to install correct aggression and not a misdirected one involving too much or uncontrolled aggression. There is a fine line between being aggressive when needed and when ethically justified and just being aggressive all the time (Boe and Ingdahl, 2017). If you deviate from basic human values and, for instance, include dehumanization and conditioning in order to kill, this may result in atrocities and war crimes. An infamous incident is the My Lai massacre in Vietnam in 1968, where United States military personnel killed hundreds of women and children (Grossman, 1995). In 2016, the Iraq Historic Allegations Team started to investigate over 1500 alleged abuses possible committed by British soldiers that had fought in the Iraq war (Brown, 2016). Understanding the soldiers’ experiences in these situations and ensuring that they get the correct education and training thus becomes extremely important. An additional element is that exposure to combat environments may affect the integration of soldiers back into the society. Learning to control aggression becomes an important element of functioning in a more normal environment (Brown, 2015).

It has been shown that the implementation of aggression and aggression control has produced good results in the education of professional soldiers in a Norwegian Army battalion (Boe and Ingdahl, 2017). Similarly, factors contributing to the willingness to kill have been identified for professional soldiers in a Norwegian Army battalion (Boe and Johannessen, 2015). Results from this study revealed that the role of the group was very important in order to increase the willingness to kill. The aggressive predisposition of the individual soldier was also found to be an important factor in order to increase the willingness to kill. However, neither the role of the leader nor the emotional distance to the enemy contributed to the soldier’s willingness to kill (Boe and Johannessen, 2015).

The American lieutenant colonel McCoy (2007) has stated that your will must be harder than anything it comes up against. During an event, your whole focus should be on the techniques, tactics, and similar things necessary to accomplish the mission. After an incident, one should normally feel relieved, grateful that one has survived, and proud (Boe, 2006). One can ask oneself whether it was these feelings that were dominant in the police officers after the incident at Utøya was over.

## A Method for Practicing Combat Mindset

Torgersen and Sæverot (2016) argue that the expertise to master unforeseen, complex, and dangerous tasks requires both realistic training and systematic reflection. The reflection phase, the so-called competence level 3 (C3 level), is essential to consolidate and strengthen experiences and new knowledge and skills that one is exposed to during training (Torgersen and Sæverot, 2016). By reflection we mean that different parts of the training sequences or scenarios are reviewed systematically and with a focus toward different areas of development. Combat mindset training (CM-training) can be one of these. All sequences are discussed and evaluated in light of CM-training, and the goal is to develop a new awareness of one’s own patterns of behavior, capacity for action, and coping skills associated with various forms of problems and stressors. Meanwhile, such a reflection phase also incorporates a common awareness in all members of the team directed toward the interaction processes during the training, where a team or several teams cooperate to solve the mission. Furthermore, the reflection should be linked to self-efficacy of sequences during training, which contained unscheduled, sudden, and unexpected events (Torgersen, 2018).

However, it is important that these reflection phases are not distanced too much from the well-known concept of “train as you fight” (Torgersen, 2008, 2015). The reflection phases should usually be associated with action-oriented and training activities closely related to the field in which one practices. Nevertheless, it may in some contexts be best to implement C3-level training as independent sequences. One example is the training and awareness of dilemmas and ethically charged situations (for instance in the form of case studies), but, in such situations, practical training should also be included. The reason for this is that it is necessary to practice the cognitive meta reflections under time pressure and stress. This systematic CM-training at the C3 level should be strengthened for all those who work with operational preparedness.

## Conclusion

The research question in the present article was “Does the police need a well-functioning combat mindset?” We discussed this question in the light of how the Norwegian Police Force handled the incident at Utøya on July 22, 2011. In this case, our conclusion is that the combat mindset of the Norwegian Police Force could have functioned better during the incident. However, when judging crises management there is always a risk for serious hindsight bias. It is easy to tell after the crisis what preparations should have been made. It is a bit trickier to tell what crisis that is going to happen next week or what preparations that is relevant in relation to future crises. The evaluation of July 22 could accordingly be a bit unfair. It is extremely difficult—and expensive—to be prepared for all possible threats. In addition, if an antagonist chooses to shoot unarmed civilians on an island, it must be considered a challenge even for a prepared police management to neutralize that threat without risking the lives and health of own personnel.

But, it is a fact that whether this had resulted in a quicker intervention against Anders Behring Breivik is impossible to know. We think, however, that a better functioning combat mindset would have resulted in quicker and improved decision making. We have tried to show that achieving a well-functioning combat mindset in general nevertheless poses a relevant challenge for an efficient and effective police force. Several techniques exist for enhancing one’s combat mindset. Positive affirmations, breathing techniques, and visualization are examples of such techniques. In addition, systematic CM-training should include both realistic training and systematic reflection to contribute to a better ability to deal with sudden and unforeseen events.

Finally, the way we have discussed the challenges in crisis management has been almost solely discussed on the individual level. We have not discussed how individual shortcomings or emotional reactions can be coped with by, e.g., group support or decision support, and this should be an interesting venue to explore when thinking of creating a correct mindset for not only the individual police man or woman but also on an organizational level.

## Author Contributions

OB and G-ET wrote the first version of the manuscript. OB, G-ET, and TS revised the manuscript and approved the final version to be published.

## Conflict of Interest

The authors declare that the research was conducted in the absence of any commercial or financial relationships that could be construed as a potential conflict of interest.
